# Unexpected Behavior of Enaminones: Interesting New Routes to 1,6-Naphthyridines, 2-Oxopyrrolidines and Pyrano[4,3,2-*de*][1,6]naphthyridines

**DOI:** 10.3390/molecules18010276

**Published:** 2012-12-27

**Authors:** Moustafa Sherief Moustafa, Saleh Mohammed Al-Mousawi, Noha Mohamed Hilmy, Yehia A. Ibrahim, Johannes C. Liermann, Herbert Meier, Mohamed Hilmy Elnagdi

**Affiliations:** 1 Department of Chemistry, Faculty of Science, University of Kuwait, P.O. Box 5969, Safat 13060, Kuwait; E-Mails: yehiaibrahim2002@yahoo.com (Y.A.I.); shelmy1941@yahoo.com (M.H.E.); 2 Women Students-Medical Studies & Sciences Sections, Department of Chemistry, College of Science, King Saud University, Riyadh, KSA, P.O. Box 22452, Riyadh 11495, Saudi Arabia; E-Mail: elnagdinoha@yahoo.com; 3 Johannes Gutenberg—Universität Mainz, Institute of Organic Chemistry, Mainz, Germany; E-Mail: hmeier@mail.uni-mainz.de

**Keywords:** enaminones, 3-amino-2-cyanopent-2-enedinitrile, 7-amino-5-oxo-5,6-dihydro-1,6-naphthyridine-8-carbonitrile, 2-aminoprop-1-ene-1,1,3-tricarbonitrile

## Abstract

Reaction of enaminones **1a**–**d** with 2-aminoprop-1-ene-1,1,3-tricarbonitrile (**2**) in the presence of AcOH/NH_4_OAc afforded 7-amino-5-oxo-5,6-dihydro-1,6-naphthyridine-8-carbonitrile derivatives **9a**–**d**. On the other hand, 2-aminopyrano[4,3,2-*de*][1,6]naphthyridine-3-carbonitriles **20a**–**c**,**e** were the only obtained products from the reactions of **1a**–**d** with **2** in the presence of AcOH/NaOAc, while **1d** afforded [3,5-bis-(4-chloro-benzoyl)-phenyl]-(4-chloro-phenyl)-methanone **21** under the same condition. The reaction of **2** with diethyl acetylenedicarboxylate in the presence of AcOH/NH_4_OAc afforded (4-cyano-5-dicyanomethylene-2-oxo-2,5-dihydro-1*H*-pyrrol-3-yl)-acetic acid ethyl ester **15B**.

## 1. Introduction

During the last decade we have been involved in a program aimed at exploring the synthetic potentials of enaminones [1] as building blocks for polyfunctionally substituted aromatics and heteroaromatics [1,2,3]. We have in the past successfully developed syntheses of polysubstituted benzenes [4,5] and polysubstituted pyridines [6,7] utilizing enaminones **1a**–**d** as starting materials. In the present article we report our further results in this area where a novel one pot synthesis of 7-amino-5-oxo-5,6-dihydro-1,6-naphthyridine-8-carbonitriles **9a**–**d**, 2-aminopyrano[4,3,2-*de*][1,6]naphthyrid-ine-3-carbonitrile derivatives **20a**–**c**,**e** and (4-cyano-5-dicyanomethylene-2-oxo-2,5-dihydro-1*H*-pyrrol-3-yl)-acetic acid ethyl ester **15B** could be achieved. To our knowledge only one derivative of the 2-aminopyrano[4,3,2-*de*][1,6]naphthyridine-3-carbonitrile system has been reported, prepared via a multistep route [8]. The newly synthesized 2-aminopyrano[4,3,2-*de*][1,6]naphthyridine-3-carbonitrile derivatives **20a**–**c**,**e** seem interesting for biological activity investigations as investigations on such polynuclear aromatics are rare [8].

## 2. Results and Discussion

**Scheme 1:**
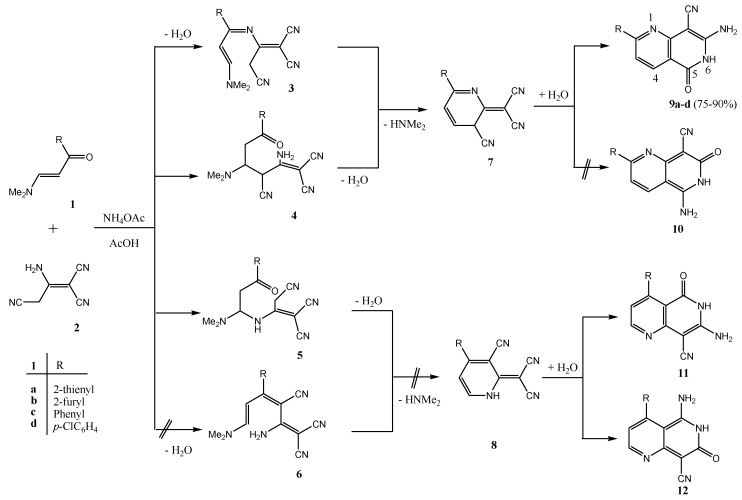
Formation of compounds **9a**–**d**.

**Figure 1:**
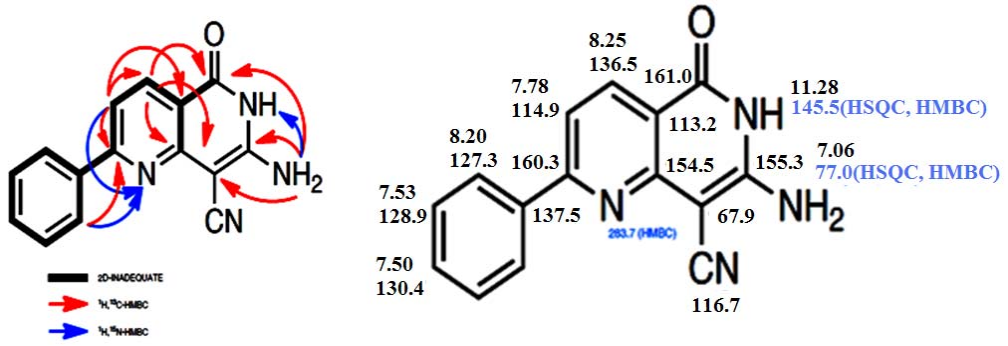
Upper part: (^13^C, ^1^H)- and (^15^N, ^1^H) couplings nJ (n = 2–4) according to the crosspeaks observed in the HMBC measurements of **9c**. Lower part: Assignment of all ^1^H, ^13^C and ^15^N signals of **9c**. The δ values obtained in CD_3_SOCD_3_ at room temperature are related to TMS and NH_3_ (liquid). The measurements were performed at 14.1 T (600 MHz for ^1^H).

**Figure 2:**
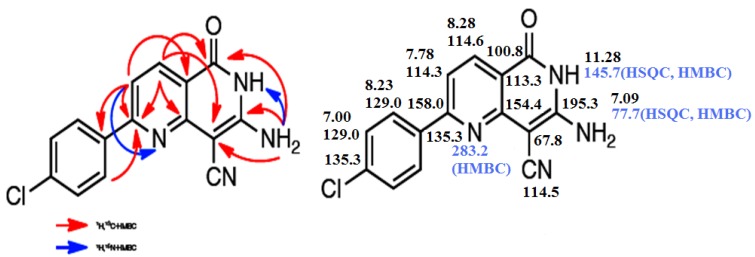
Upper part: (^13^C, ^1^H)- and (^15^N, ^1^H) couplings nJ (n = 2–4) according to the crosspeaks observed in the HMBC measurements of **9d**. Lower part: Assignment of all ^1^H, ^13^C and ^15^N signals of **9d**. The δ values obtained in CD_3_SOCD_3_ at room temperature are related to TMS and NH_3_ (liquid). The measurements were performed at 14.1 T (600 MHz for ^1^H).

Although the reaction of enaminone **1c** and 3-amino-2-cyanopent-2-enedinitrile (**2**) in refluxing acetic acid in presence of ammonium acetate (NH_4_OAc) has been reported earlier [1,9] to afford 2,4-diamino-5-benzoylisophthalonitrile, with molecular formula C_15_H_10_N_4_O and molecular mass M^+^ = 262, we found, however, that enaminone **1c** and compound **2** react in acetic acid in the presence of NH_4_OAc to yield a completely different product with the same molecular formula and molecular mass. Both starting compounds the enaminone **1** and 3-amino-2-cyanopent-2-enedinitrile (**2**) are bifunctional reactants. The carbonyl group of **1** can form an imine (**1** + **2**→ **3**) or participate in a Knoevenagel reaction with the methylene group of **2** (**1** + **2**→ **6**). Michael-type additions **1**+**2**→ **4** or **1** + **2**→ **5** are other alternatives. The subsequent ring closure reactions can lead to the pyridine derivatives **7** or **8**. Finally, the 1,6-naphthyridine systems **9**–**12** can also be generated. The yields obtained from **1a**–**d** are between 75 and 90%. We have depicted all possible end products that could be obtained from reacting **1a**–**d** and **2** in Scheme 1 as secondary carbamides, but their tautomers having a hydrogen atom bound to N-1 or to the oxygen atom have to be considered as well. The structure determination of the reaction products of **1a**–**d** and 3-amino-2-cyanopent-2-enedinitrile (**2**) proved to be very difficult, because all twelve possible 1,6-naphthyridine derivatives should have similar ^1^H- and ^13^C-NMR spectra. Therefore, we performed a series of 2D NMR measurements: (^1^H,^1^H)COSY, (^1^H,^13^C)HSQC, (^1^H,^13^C)HMBC, (^1^H,^15^N)HSQC, (^1^H,^15^N)HMBC, and INADEQUATE. The final decision was made in favor of the structures **9a**–**d**. Figure 1 and Figure 2 showed the ^1^H, ^13^C and ^15^N chemical shifts and the results of the 2D-INADEQUATE and the two HMBC measurements which represent the basis for the assignment of the chemical shifts (Scheme 1).

**Figure 3:**
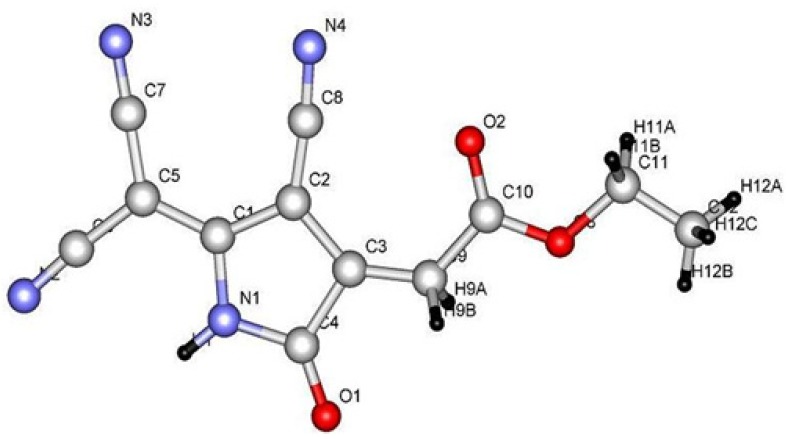
X-ray crystal structure of compound **15A**.

**Scheme 2:**
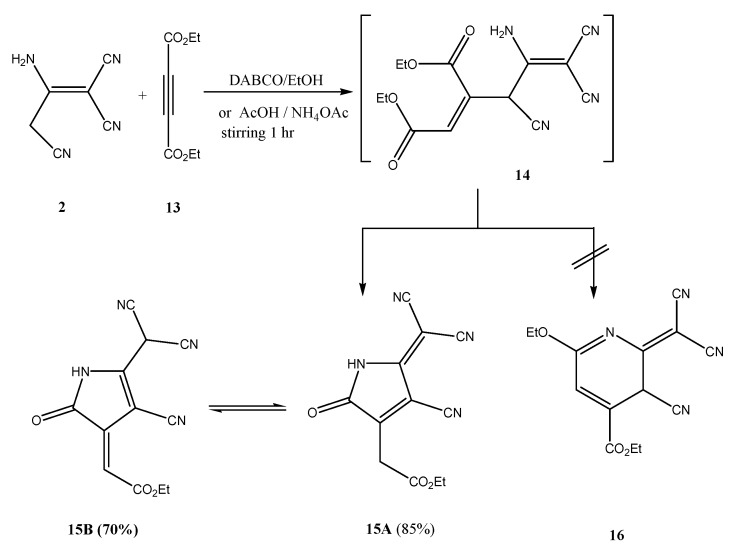
Formation of compound **15B**.

In order to extend this finding further we reacted 3-amino-2-cyanopent-2-enedinitrile (**2**) with diethyl acetylenedicarboxylate (**13**) in the presence of AcOH/NH_4_OAc. In this case, however, ethyl (4-cyano-5-dicyanomethylene-2-oxo-pyrrolidin-3-ylidene) acetate **15B** was obtained as indicated by an X-ray crystal structure determination (Figure 3) [10]. It is believed that compound **2 **reacts with **13** to initially afford adduct **14** that cyclizes preferably to the pyrrolidine **15** rather than the alternative pyridine derivative **16** (Scheme 2). Although **15** has been shown to exist as a solid, in DMSO solution only form **15B** exists, as indicated by the ^1^H-NMR data that showed a singlet at δ = 5.50 ppm for the methylene proton and a broad signal at δ = 2.48 ppm for proton of the dicyanomyl moiety.

**Scheme 3:**
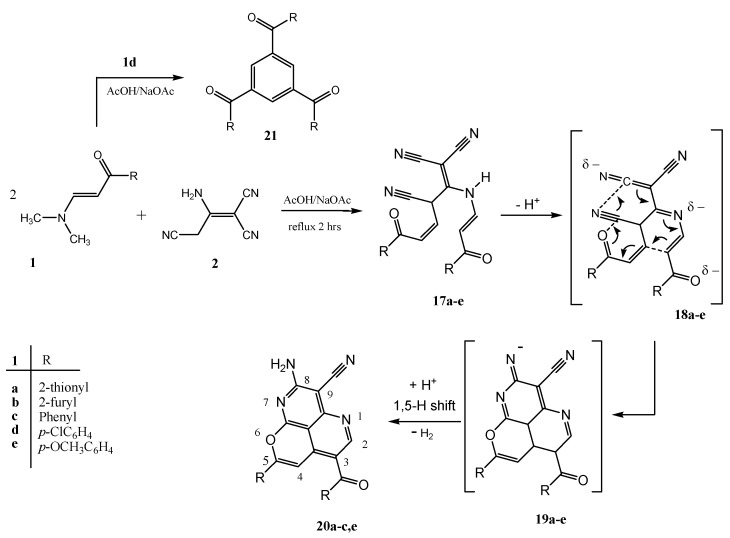
Formation of compounds **20a**–**c**,**e**.

We conducted the same reactions of enaminones **1a**–**c**,**e** with 3-amino-2-cyanopent-2-enedinitrile (**2**) in the presence of AcOH/NaOAc. This reaction afforded in this case a different product with molecular formula C_20_H_10_N_4_O_2_S_2_ and molecular mass M^+^= 402. It is believed that compound **1a** reacted with 3-amino-2-cyanopent-2-enedinitrile (**2**) to form the highly unsaturated intermediates **17a**–**c**,**e** and their anions **18a**–**c**,**e**, respectively (Scheme 3).

The intermediate **18** can undergo a cyclic π-electron shift (6π → 3σ + 3π, valence isomerization) to **19a**–**c**,**e**. Protonation of **19a**–**c**,**e**, followed by 1,5-H-shift and dehydrogenation lead finally to the 2-aminopyrano[4,3,2-*de*][1,6]naphthyridine-3-carbonitrile **20a**–**c**,**e**. Yields of **20a**–**c**,**e** amounted to 85–92% when 2:1-mixtures of **1** and **2** were refluxed in AcOH/NaOAc. The reaction of **1d** with 2 under the same conditions afforded [3,5-bis-(4-chloro-benzoyl)-phenyl]-(4-chloro-phenyl)-methanone **21** previously obtain by upon refluxing **1d** in AcOH. It has been previously observed that **1d** readily trimerise on attempted condensation with nucleophils [12].

**Figure 4:**
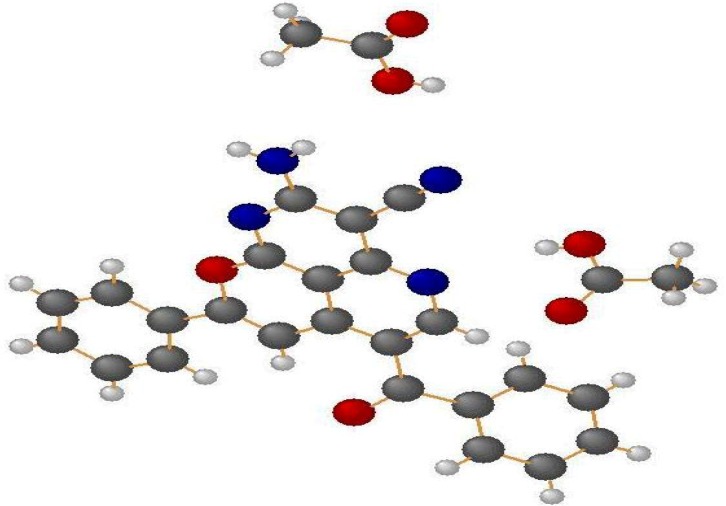
Crystal structure of **20c**.

**Figure 5:**
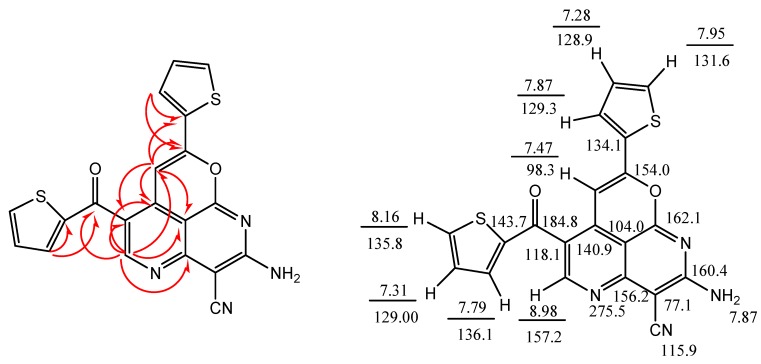
Left part: (^13^C,^1^H) couplings ^n^*J* (n = 2–4) according to the crosspeaks observed in HMBC measurement of **20a**. Right part: Assignment of all ^1^H and ^13^C signals and one ^15^N signal of **20a**. The δ values obtained in CD_3_SOCD_3_ at room temperature are related to TMS and NH_3_ (liquid). The measurements were performed at 14.1 T (600 MHz for ^1^H).

The structure determination of **20** was based on 2D NMR measurements (COSY, HSQC, HMBC and (^1^H, ^15^N) HMBC) of **20a** and on a crystal structure analysis of **20c** (Figure 4 and Figure 5) [11].

## 3. Experimental

### 3.1. General

Melting points are reported uncorrected and were determined with a Sanyo (Gallaenkamp) instrument. Infrared spectra were recorded using KBr pellets and a Perkin-Elmer 2000 FT–IR instrument. ^1^H- and ^13^C-NMR spectra were determined by using a Bruker DPX instrument at 600 MHz for ^1^H-NMR and 150 MHz for ^13^C-NMR and either CDCl_3_ or DMSO-d_6_ solutions with TMS as internal standards. Chemical shifts are reported in δ (ppm). Mass spectra were measured using VG Autospec Q MS 30 and MS 9 (AEI) spectrometer, with the EI (70 EV) mode. Elemental analyses were carried out by using a LEOCHNS-932 Elemental Analyzer. X-ray crystal structure determined by using a Single Crystal X-ray Crystallography-Rigaku Rapid II & Bruker X8 Prospector system.

### 3.2. General Procedure for the Synthesis of ***9a–d***

A mixture of enaminone **1a**–**d** (0.01 mol) and 3-amino-2-cyanopent-2-enedinitrile (**2**, 1.32 g, 0.01 mol) in AcOH (25 mL)/NH_4_OAc (1 g) was heated under reflux for 2 h (followed until completion by TLC using 1:1 ethyl acetate–petroleum ether as eluent). The mixture was then cooled and poured onto ice-water. The solid, so formed, was collected by filtration and recrystallized from AcOH to give yellow crystals.

*7-Amino-5-oxo-2-(thienyl)-5,6-dihydro-1,6-naphthyridine-8-carbonitrile* (**9a**). Yield 75%; mp. 291–292 °C. Anal. Calcd. for C_13_H_8_N_4_OS (268.29): C, 58.20; H, 3.01; N, 20.88; S, 11.95%. Found: C, 58.17; H, 3.13; N, 20.65; S, 11.92%; IR (KBr, cm^−1^): 3471 (NH), 3363, 3295 (NH_2_) 2252 (CN), 1652 (CO); ^1^H-NMR (DMSO-d_6_): δ = 7.05 (br, 2H, NH_2_, D_2_O exchangeable), 7.21 (t, 1H, *J* = 4.0, thienyle-H), 7.69 (d, 1H, *J* = 8.0, CH), 7.77 (d, 1H, *J* = 4.0, thienyl-H), 7.94 (d, 1H, *J* = 4.0, thienyl-H), 8.22 (d, 1H, *J* = 8.0, CH), 11.22 (br, 1H, NH, D_2_O exchangeable); ^13^C-NMR (DMSO-d_6_): δ = 160.6, 156.1, 155.2, 154.4, 143.6, 136.2, 130.9, 128.6, 127.7, 116.2, 113.5, 112.56, 67.4. MS: *m/z* (%) 268 (M^+^, 40), 256 (35), 241 (15), 213 (25), 185 (25), 169 (20), 129 (55), 97 (40), 73 (100).

*7-Amino-2-(furyl)-5-oxo-5,6-dihydro-1,6-naphthyridine-8-carbonitrile* (**9b**). Yield 80%; mp. 287–289 °C. Anal. Calcd. for C_13_H_8_N_4_O_2_ (252.23): C, 61.90; H, 3.20; N, 22.21%. Found: C, 61.84; H, 3.31; N, 22.32%; IR (KBr, cm^−1^): 3424 (NH), 3343, 3240 (NH_2_), 2214 (CN), 1662 (CO); ^1^H-NMR (DMSO-d_6_): δ = 6.72 (t, 1H, *J* = 4.0, furyl-H), 7.05 (br, 2H, NH_2_, D_2_O exchangeable), 7.25 (d, 1H, *J* = 4.0, furyl-H), 7.51 (d, 1H, *J* = 8.0, CH), 7.94 (d, 1H, *J* = 4.0, furyl-H), 8.25 (d, 1H, *J* = 8.0, CH), 11.24 (br, 1H, NH, D_2_O exchangeable); ^13^C-NMR (DMSO-d_6_): δ = 160.1, 155.3, 154.5, 152.4, 152.3, 145.7, 136.3, 116.4, 113.3, 112.7, 112.6, 111.9, 67.5. MS: *m/z* (%) 252 (M^+^, 100), 224 (20), 195 (10), 73 (90).

*7-Amino-5-oxo-2-phenyl-5,6-dihydro-1,6-naphthyridine-8-carbonitrile* (**9c**). Yield 90%; mp. 253–255 °C. Anal. Calcd. for C_15_H_10_N_4_O (262.27): C, 68.69; H, 3.84; N, 21.36%. Found: C, 68.65; H, 3.75; N, 21.40%; IR (KBr, cm^−1^): 3325 (NH), 3251, 3209 (NH_2_), 2209 (CN), 1673 (CO); ^1^H-NMR (DMSO-d6): δ = 7.07 (br, 2H, NH_2_, D_2_O exchangeable), 7.52–7.53 (m, 3H, Ph-H), 7.75 (d, 1H, *J* = 8.0, CH), 8.20–8.21 (m, 2H, Ph-H), 8.28 (d, 1H, *J* = 8.0, CH), 11.27 (br, 1H, NH, D_2_O exchangeable); ^13^C-NMR (DMSO-d_6_): δ = 160.8, 160.2, 155.2, 154.3, 137.5, 136.4, 130.3, 128.8 (2C), 127.2 (2C), 116.5, 114.8, 113.1, 67.8. MS: *m/z* (%) 262 (M^+^, 100), 234 (15), 217 (10), 192 (5), 164 (10), 129 (10), 83 (10), 73 (25).

*7-Amino-2-(4-chlorophenyl)-5-oxo-5,6-dihydro-1,6-naphthyridine-8-carbonitrile* (**9d**). Yield 90%; mp. 275–276 °C. Anal. Calcd. for C_15_H_9_ClN_4_O (296.72): C, 60.72; H, 3.06; N, 18.88%. Found: C, 60.70; H, 3.11; N, 18.87%; IR (KBr, cm^−1^): 3436 (NH), 3324, 3216 (NH_2_), 2211 (CN), 1678 (CO); ^1^H-NMR (DMSO-d_6_): δ = 7.06 (s, 2H, NH_2_, D_2_O exchangeable), 7.54 (d, 2H, Ph-H), 7.69 (d, 1H, *J* = 8.0, CH), 8.17 (d, 2H, Ph-H), 8.23 (d, 1H, *J* = 8.0, CH), 11.25 (br, 1H, NH, D_2_O exchangeable); ^13^C-NMR (DMSO-d_6_): δ = 160.7, 158.8, 155.2, 154.2, 136.4, 136.2, 135.2, 129.0 (2C), 128.8 (2C), 116.4, 114.6, 113.2, 67.8. MS: *m/z* (%) 296 (M^+^, 100), 268 (20), 216 (15), 189 (15), 164 (10), 130 (10), 88 (15), 73 (20).

*Synthesis of (4-cyano-5-dicyanomethylene-2-oxo-2,5-dihydro-1H-pyrrol-3-yl)-acetic acid ethyl ester* (**15B**). A mixture of 3-amino-2-cyanopent-2-enedinitrile (**2**, 1.32 g, 0.01 mol) and diethyl acetylenedicarboxylate (**13**, 1.70 g, 0.01 mol) in AcOH (25 mL)/NH_4_OAc (1 g) was refluxed for 2 h (followed until completion by TLC using 1:1 ethyl acetate–petroleum ether as eluent). The mixture was cooled and then was poured onto ice-water. The solid, so formed, was collected by filtration and recrystallized from EtOH to give orange crystals, yield 70%; mp. 200–202 °C. Anal. Calcd. for C_12_H_8_N_4_O_3_ (256.22): C, 56.25; H, 3.15; N, 21.87%. Found: C, 56.22; H, 3.27; N, 22.00%; IR (KBr, cm^−1^): 3363 (NH), 2223 (CN), 2211 (2CN), 1695 (CO), 1653 (CO); ^1^H-NMR (DMSO-d_6_): δ = 1.22 (t, 3H, *J* = 8.0, CH_3_), 2.48 (br, 1H, , *J* = 8.0, CH), 4.10 (q, 2H, CH_2_), 5.50 (s, 1H, CH), 10.84 (br, 1H, NH, D_2_O exchangeable); ^13^C-NMR (DMSO-d_6_): δ = 169.0, 165.8, 161.3, 138.5, 117.2, 116.0, 115.6, 100.1 (2C), 68.2, 59.0, 14.2. MS: *m/z* (%) 256 (M^+^, 50), 211 (15), 184 (75), 156 (10), 112 (100), 97 (10), 84 (25), 70 (25), 55 (90).

### 3.3. General Procedure to Syntheses of ***20a–c,e***

A mixture of enaminone **1a**–**e** (0.01 mol) and 2-aminoprop-1-ene-1,1,3-tricarbonitrile (**2**, 1.32 g, 0.01 mol) in AcOH/NaOAc (1 g) was refluxed for 2 h (followed until completion by TLC using 1:1 ethyl acetate–petroleum ether as eluent). The mixture was cooled and then was poured onto ice-water. The solid, so formed, was collected by filtration and recrystallized from AcOH to give yellow crystals. 

*2-Amino-8-(thiophen-2-yl)-6-(thiophene-2-carbonyl)pyrano-[4,3,2-de][1,6]naphthyridine-3-carbonitrile* (**20a**). Yield 92%; mp. 369–370 °C. Anal. Calcd. for C_20_H_10_N_4_O_2_S_2_ (402.45): C, 59.69; H, 2.50; N, 13.92; S, 15.93%. Found: C, 59.72; H, 2.33; N, 13.97; S, 15.86%; IR (KBr, cm^−1^): 33363, 3295 (NH_2_), 2213 (CN), 1642 (CO); ^1^H-NMR (DMSO-d_6_): δ = 7.27 (t, 1H, *J* = 4.0, thienyl-H), 7.31 (t, 1H, *J* = 4.0, thienyl-H), 7.47 (s, 1H, CH), 7.78 (d, 1H, *J* = 4.0, thienyl-H), 7.86 (br, 3H, thienyl-H, NH_2_, D_2_O exchangeable), 7.94 (d, 1H, *J* = 4.0, thienyl-H), 8.16 (d, 1H, *J* = 4.0, thienyl-CH), 8.98 (s, 1H, CH); ^13^C-NMR (DMSO-d_6_): δ = 184.8, 162.1, 160.4, 157.2, 156.2, 154.0, 143.7, 136.1, 135.8, 134.1 (2C), 131.6, 129.3, 129.00, 128.9, 118.1, 115.9, 104.0, 98.3, 77.1 . MS: *m/z* (%) 402 (M^+^, 100), 373 (15), 319 (25), 263 (5), 236 (5), 187 (10), 111 (30), 83 (5).

*2-Amino-6-(furan-2-carbonyl)-8-(furan-2-yl)pyrano[4,3,2-de]-[1,6]naphthyridine-3-carbonitrile* (**20b**). Yield 90%; mp. 375–377 °C. Anal. Calcd. for C_20_H_10_N_4_O_4_ (370.32): C, 64.87; H, 2.72; N, 15.13%. Found: C, 64.88; H, 2.68; N, 15.22%; IR (KBr, cm^−1^): 3471, 3373(NH_2_), 2210 (CN), 1653 (CO); ^1^H-NMR (DMSO-d_6_): δ = 6.78 (t, 1H, *J* = 4.0, furyl-H), 6.82 (t, 1H, *J* = 4.0, furyl-H), 7.19 (d, 1H, *J* = 4.0, furyl-H), 7.44 (s, 1H, CH), 7.46 (d, 1H, *J* = 4.0, furyl-H), 7.83 (br, 2H, NH_2_, D_2_O exchangeable), 8.04 (d, 1H, *J* = 4.0, furyl-H), 8.24 (d, 1H, *J* = 4.0, furyl-H), 8.98 (s, 1H, CH); ^13^C-NMR (DMSO-d_6_): δ = 179.20, 162.29, 161.98, 160.13, 157.31, 156.08, 151.64, 150.06, 148.72, 147.23, 145.30, 140.47, 121.34, 117.48, 115.76, 113.36, 112.97, 103.98, 97.56, 77.23. MS: *m/z* (%) 370 (M^+^, 90), 264 (15), 224 (25), 195 (15), 169 (10), 129 (10), 83 (30), 73 (35).

*2-Amino-6-benzoyl-8-phenylpyrano[4,3,2-de][1,6]naphthpyrid-ine-3-carbonitrile* (**20c**). Yield 88%; mp. 318–319 °C. Anal. Calcd. for C_24_H_14_N_4_O_2_ (390.11): C, 73.84; H, 3.61; N, 14.35%. Found: C, 73.92; H, 3.6; N, 14.28%; IR (KBr, cm^−1^): 3445, 3341 (NH_2_), 2216 (CN), 1646 (CO); ^1^H-NMR (DMSO-d_6_): δ = 7.56–7.61 (m, 5H, Ph-H), 7.65 (s, 1H, CH), 7.69–7.86 (m, 7H, Ph-H, NH_2_, D_2_O exchangeable), 8.72 (s, 1H, CH); ^13^C-NMR (DMSO-d_6_): δ = 193.50, 162.10, 160.86, 158.22, 158.31, 156.20, 141.80, 141.10, 137.70, 135.20, 132.90, 131.60, 130.70, 129.60 (2C), 129.30, 128.80 (2C), 125.70, 117.75, 115.77, 104.21, 100.10, 77.10. MS: *m/z* (%) 390 (M^+^, 100), 373 (15), 313 (65), 257 (10), 230 (10), 188 (5), 181 (5), 105 (20), 77 (25).

*2-Amino-6-(4-methoxybenzoyl)-8-(4-methoxyphenyl)pyrano-[4,3,2-de][1,6]naphthyridine-3-carbonitrile* (**20e**). Yield 85%; mp. 325–327 °C. Anal. Calcd. for C_26_H_18_N_4_O_4_ (450.45): C, 69.33; H, 4.03; N, 12.44%. Found: C, 69.40; H, 4.12; N, 12.42%; IR (KBr, cm^−1^): 3424, 3343 (NH_2_), 2214 (CN), 1644 (CO); ^1^H-NMR (DMSO-d_6_): δ = 3.84 (s, 3H, CH_3_), 3.88 (s, 3H, CH_3_), 7.07-7.11 (m, 4H, Ph-H), 7.42 (s, 1H, CH), 7.75–7.80 (m, 6H, Ph-H, NH_2_, D_2_O exchangeable), 8.69 (s, 1H, CH); ^13^C-NMR (DMSO-d_6_): δ = 191.90, 163.30, 162.90, 160.82, 158.21, 157.65, 157.50, 156.20, 140.81, 132.11 (2C), 130.16 (2C), 127.50, 123.10, 118.62, 115.53, 114.27 (2C), 114.10 (2C), 103.90, 98.50, 77.15, 55.36, 55.41. MS: *m/z* (%) 450 (M^+^, 100), 419 (20), 407 (5), 343 (30), 300 (10), 211 (15), 135 (25), 107 (5), 77 (20).

### 3.4. Synthesis of [3,5-bis-(4-chlorobenzoyl)phenyl]-(4-chlorophenyl)methanone ***(21)***

A mixture of enaminone **1d** (2.09 g, 0.01 mol) and 2-aminoprop-1-ene-1,1,3-tricarbonitrile (**2**, 1.32 g, 0.01 mol) in AcOH/NaOAc (1 gm) was refluxed for 2 h (followed until completion by TLC using 1:1 ethyl acetate–petroleum ether as eluent). The mixture was cooled and then was poured onto ice-water. The solid, so formed, was collected by filtration and recrystallized from AcOH to give faint yellow crystals, yield 80%. This product was also prepared *via* refluxing **1d** in AcOH as described earlier by Elnagdi *et al.* [12]. 

## 4. Conclusions

New simple and efficient routes for the synthesis of 7-amino-5-oxo-5,6-dihydro-1,6-naphthyridine-8-carbonitrile derivatives **9a**–**d**, (4-cyano-5-dicyanomethylene-2-oxo-pyrrolidin-3-ylidene)-acetic acid ethyl ester **15B** and 2-aminopyrano[4,3,2-*de*][1,6]naphthyridine-3-carbonitrile derivatives **20a**–**c**,**e** from the reaction of enaminones with 2-aminoprop-1-ene-1,1,3-tricarbonitrile (**2**) have been described. These products look interesting for potential biological evaluation. Moreover, all the products have latent functional moieties that seem interesting precursors to other derivatives of the described ring systems. 

## References

[1] Al-Mousawi S.M., Moustafa M.S., Elnagdi M.H. (2011). Green synthetic approaches: solventless synthesis of polyfunctionally substituted aromatics as potential versatile building blocks in organic synthesis utilizing enaminones and enaminonitriles as precursors. Green Chem. Lett. Rev..

[2] Al-Zaydi K.M., Borik R.M., Mekheimer R.A., Elnagdi M.H. (2010). Green chemistry: A facile synthesis of polyfunctionally substituted thieno[3,4-c]pyridinones and thieno[3,4-d]pyridazinones under neat reaction conditions. Ultrasonics Sonochem..

[3] Al-Mousawi S.M., Moustafa M.S., Elnagdi M.H. (2009). A novel synthesis of 2-arylhydrazono-6-amino-4-arylbenzene-1,3-dicarbonitriles and their conversion into phthalazines. Tetrahedron Lett..

[4] Al-Awadi N.A., Abdelkhalik M.M., Abdelhamid I.A., Elnagdi M.H. (2007). Pyrolytic methods in organic synthesis: Novel routes for the synthesis of 3-oxoalkanenitriles, 2-acyl anilines, and 2-aroyl anilines. Synlett.

[5] Al-Saleh B., Abdelkhalik M.M., Eltoukhy A.M., Elnagdi M.H. (2002). Enaminones in heterocyclic synthesis: A new regioselective synthesis of 2,3,6-trisubstituted pyridines, 6-substituted-3-aroylpyridines and 1,3,5-triaroylbenzenes. J. Heterocycl. Chem..

[6] Al-Mousawi S.M., Moustafa M.S., Abdelhamid I.A., Elnagdi M.H. (2011). Reassignment of the structures of condensation products of α-keto α'-formylarylhydrazones with ethyl cyanoacetate: A novel route to ethyl 5-arylazo-2-hydroxynicotinates. Tetrahedron Lett..

[7] Riyadh S.M., Abdelhamid I.A., Al-Matar H.M., Hilmy N.M., Elnagdi M.H. (2008). Enamines as precursors to polyfunctional heteroaromatic compounds; A decade of development. Heterocycles.

[8] VanAllan J.A., Petropoulos C.C., George A., Maier D.P. (1970). Reactions of some 4-dicyanomethylenepyran derivatives with malononitrile and hydrazines. J. Heterocycl. Chem..

[9] Helmy N.M., El-Baih F.E.M., Al-Alshaikh M.A., Moustafa M.S. (2011). A route to dicyanomethylene pyridines and substituted benzonitriles utilizingmalononitrile dimer as a precursor. Molecules.

[B10-molecules-18-00276] 10.CCDC 861196 contains the supplementary crystallographic data for compound **15A**. These data can be obtained free of charge from the Cambridge Crystallographic Data Centre *via* www.ccdc.cam.ac.uk.

[B11-molecules-18-00276] 11.CCDC 838314 contains the supplementary crystallographic data for compound **20c**. These data can be obtained free of charge from the Cambridge Crystallographic Data Centre *via* www.ccdc.cam.ac.uk

[12] Al-Enezi A., Al-Saleh B., Elnagdi M.H. (1997). Studies with heteroaromatic amines: The reaction of some heteroaromatic amines with 1-substituted 3-dimethylaminopropanones, Enaminones and cinnamonitriles. J. Chem. Res. (S).

